# Assessment of Reference Values for Copper and Zinc in Blood Serum of First and Second Lactating Dairy Cows

**DOI:** 10.4061/2010/194656

**Published:** 2010-03-30

**Authors:** Markus Spolders, Martin Höltershinken, Ulrich Meyer, Jürgen Rehage, Gerhard Flachowsky

**Affiliations:** ^1^Institute of Animal Nutrition, Friedrich-Loeffler-Institute (FLI)—Federal Research Institute for Animal Health, Bundesallee 50, 38116 Braunschweig, Germany; ^2^Clinic for Cattle, University of Veterinary Medicine Hanover, Bischofsholer Damm 15, 30173 Hannover, Germany

## Abstract

The influence of different copper and zinc contents in rations on blood serum concentrations in dependence on feeding (Groups A and B) and lactation (Lactation 1 and 2) was tested in a feeding trial with 60 German Holstein cows. All animals received a diet based on maize and grass silage ad libitum. 30 cows received a concentrate supplemented with copper and zinc as recommended (Group A), whereas the other 30 animals were offered a concentrate with roughly double the amount of copper and zinc (Group B). Blood samples were taken several times during the lactation to analyse serum concentrations of copper and zinc. Copper serum concentration was influenced neither by the different feeding (11.7 *μ*mol/L in Group A and 12.3 *μ*mol/L in Group B) nor by the lactation (12.0 *μ*mol/L in Lactation 1 and 12.1 *μ*mol/L in Lactation 2). Zinc serum concentration was significantly influenced as well as by feeding (14.1 *μ*mol/L in Group B and 12.5 *μ*mol/L in Group A) and lactation (14.2 *μ*mol/L in the second lactation and 12.8 *μ*mol/L for first lactating cows). For an exact diagnosis of trace element supply, blood serum is a not qualified indicator; other sources (feedstuffs, liver, hair) must also be investigated.

## 1. Introduction

Copper and zinc are essential trace elements and they are involved in many physiological processes in animals [1–4]. Blood samples are frequently taken for analyses to examine the supply with copper and zinc in practice. The measured trace element concentrations are compared with so-called reference values to characterise the trace element supply and the status of the animals (deficient, sufficient, overdosed). Such a reference value is defined as a quantitative parameter, which is derived under exactly defined conditions from a group of adequately characterized probands with a definite mathematical-statistical method [5]. Currently, the variation between different reference values for copper and zinc in blood serum of ruminants in the literature [6] is very high (Table 1), although the methods of their calculation are internationally standardised [7, 8]. 

However, for some reference values, the method of determination is not described in detail, and the application of the suggested calculation methods could not be reproduced [9]. In practice, for many reference values, neither the origin nor the history of the calculation procedures are known. Consequently, use of them in practice is really difficult, because the applied reference values should be determined under comparable field conditions such as the actual analysed values. Reference values are only appropriate as a theoretical orientation and could not be evaluated in the same way for different animals [10, 11]. In the last ten years, there is an increasing reporting in veterinary practice about a “trace element deficiency without clinical signs” diagnosed only by serum trace element concentrations below the applied reference values. But what are the reasons for these discrepancies—are serum samples not qualified for diagnosing trace element supply or must the reference values be adapted to actual feeding conditions? The aim of the present study was to investigate the influences of different copper and zinc supply on copper and zinc concentrations in the blood serum. The measured values were used to calculate reference values for the different feeding groups and lactations, and to compare this with specifications in the literature. 

## 2. Material and Methods

### 2.1. Animals

Experiments were conducted with a total of 60 dairy cows (38 first lactating and 22 second lactating cows) of the German Holstein breed. At the beginning of the experiment (after calving), the 60 animals were divided into two feeding groups (*n* = 30) with 19 first lactating and 11 second lactating cows in each feeding group. The total length of the experimental feeding comprised 300 days of lactation.

### 2.2. Feeding

All animals received a mixture of maize silage and grass silage in a ratio of 60-to-40 percent on dry matter (DM) basis. The roughage was fed ad libitum in computerized weight troughs. The amount of roughage intake was expected to supply nutrients and energy for a milk yield of 10 kg fat corrected milk (FCM) per day. The feed intake of each animal on each visit to the trough was recorded individually. The concentrate was fed to the animals with automatic concentrate feeders according to their milk yield. The concentrate portion was 0.5 kg per additional kg of milk. The main difference between the two feeding groups was the different content of copper and zinc in the concentrate. In Group A, the cows were fed a concentrate with a copper and zinc content according to the recommendations of the GfE (10 mg Cu and 50 mg Zn/kg DM [12]), whereas the animals in Group B received nearly double the recommended amounts of copper and zinc. The copper and zinc contents of the different feed components and the total rations are listed in Table 2.

The energy content (NEL) and the utilizable crude protein content (uCP) were in accordance with the recommendations of the GfE [12] for both groups. The only difference between the two feeding groups (A and B) was the different copper and zinc supply in the two concentrates.

### 2.3. Data Collection, Samples, and Analyses

Individual dry matter intake of each animal was recorded with computerized weight troughs for roughage and with automatic feeders for concentrate. For each animal, the total dry matter (roughage and concentrate) and trace element intake (copper and zinc) were summarized each day. At six specified times during the lactation (Days 0, 7, 28, 56, 112, and 252), blood samples of all animals were taken from the jugular vein at the same time of the day (one hour after feeding) to exclude any diurnal effects. The concentration of copper and zinc in the serum was analysed in the laboratory of the Clinic for Cattle of the University of Veterinary Medicine in Hanover with inductively-coupled-plasma optical-emission-spectrometry (ICP-OES), a common used analysing method for trace elements. 

### 2.4. Statistical Analysis

The statistical analysis was conducted using the software package SAS (SAS Institute, Version 9.1, 2003). Feed intake, copper and zinc intake, and the serum samples were analysed using the general linear model procedure of SAS. A one-way factorial design of ANOVA was used for the different proved variables “group” and “lactation” and had the following model:

(1) $$y_{ij}=\mu +\alpha_{i}+e_{ij},$$

where *y*_*ij*_ = tested parameter of the cow “*j*” fed diet type “*i*”, *μ* = overall mean, *α*_*i*_ = effect of group (A and B) or lactation (1 and 2), *e*_*ij*_ = error term. Mean value differences were tested with a multiple *t*-test (Tukey). The differences were considered statistically significant if *P* < .05. Reference values for copper and zinc in blood serum were calculated by the nonparametric method as 95 percent percentile and by the parametric method as mean value ±2 × standard deviation for the two feeding groups (A and B) and lactations (1 and 2). All analysed serum concentrations during the whole experimentation (six sampling times) are involved in the calculation of reference values (*n* = 170 blood samples for Group A and *n* = 172 for Group B, *n* = 212 for Lactation 1 and *n* = 130 for Lactation 2). These reference values are population-based reference ranges, because they are based on repeated measurements of the same individuals. Additionally, the calculated reference values were compared with different reference values given in the literature.

## 3. Results

### 3.1. Dry Matter, Copper, and Zinc Intake

The mean dry matter intake was not significantly influenced by the feeding group, whereas there is a significant influence of lactation number (Table 3). The higher copper and zinc supply in Group B resulted in a mean dry matter intake of 17.4 ± 3.2 kg/d, which is not significantly different from the mean DM-intake of the animals in Group A (17.3 ± 3.3 kg/d). However, the mean dry matter intake of cows in the second lactation (19.1 ± 3.1 kg/d) was significantly higher than that of first lactating cows (16.4 ± 2.9 kg/d). 

The mean copper and zinc intake was significantly higher for cows in Group B (395 ± 78 mg Cu/d and 1993 ± 397 mg Zn/d) as a main consequence of the higher content in the concentrate for Group B in comparison to the copper and zinc intake of the animals in Group A (193 ± 39 mg Cu/d and 1041 ± 215 mg Zn/d). The lactation number did not significantly influence the copper and zinc intake (Table 3). The tendentially higher intakes in the second lactation resulted from the significantly higher DM-intake.

### 3.2. Copper and Zinc Concentration in Blood Serum

**Figure 1:**
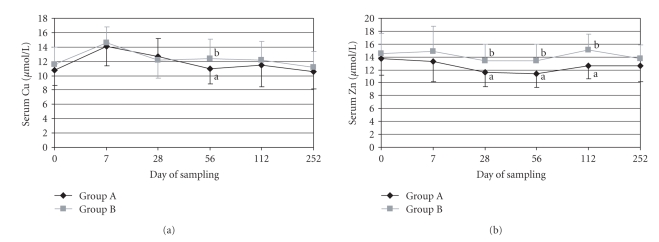
Copper (a) and zinc concentration (b) in blood serum (*μ*mol/L) in the different feeding groups (Groups A and B); a < b, *P* < .05.

The different copper supply between the animals of Groups A and B had no significant influence on the serum copper concentration (Figure 1(a)). In both feeding groups, the highest serum copper concentration was analysed seven days after starting (14.1 and 14.5 *μ*mol/L); during the course of the experimentation the serum copper concentration decreased slightly to values of 10.5 *μ*mol/L for Group A and 11.2 *μ*mol/L for Group B, which are lower than the reference ranges for copper (12–24 *μ*mol/L [19]). The mean copper concentrations also did not significantly differ between feeding groups (11.7 *μ*mol/L in Group A and 12.3 *μ*mol/L in Group B). 

In contrast to copper, the serum zinc concentration (Figure 1(b)) was significantly influenced by the different zinc supply. During the whole experimental feeding the mean serum zinc concentration was significantly higher for the animals in Group B (14.1 *μ*mol/L), compared to the serum zinc concentration of the cows in Group A (12.5 *μ*mol/L). Only the analysed serum zinc concentrations in Group B are in agreement with the reference ranges for zinc (12–24 *μ*mol/L [19]), whereas the majority of all analysed serum zinc concentrations in Group A were lower than 12 *μ*mol/L during the whole sampling period (day 0–252).

**Figure 2:**
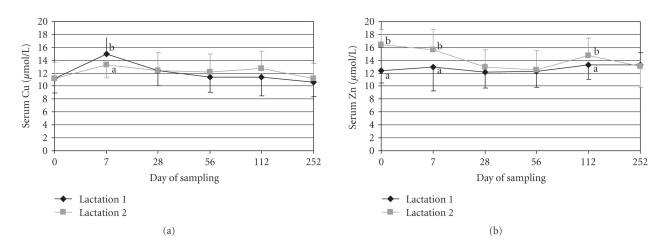
Copper (a) and zinc concentration (b) in blood serum (*μ*mol/L) in the different lactations (1 and 2); a < b, *P* < .05.

The analysed copper concentrations in blood serum were nearly constant during the whole experimental period and there were no significant differences between the two lactations (12.0 *μ*mol/L in Lactation 1 and 12.1 *μ*mol/L in Lactation 2 on average, Figure 2(a)). Seven days after starting the different copper supply in Lactation 1, the serum copper concentration was highest (15.0 *μ*mol/L) and decreased slightly till the end of the experimentation (10.6 *μ*mol/L on day 252). In the second lactation, the copper concentration was nearly constant (11.2–13.3 *μ*mol/L), the increase seven days after beginning of the experimental feeding was only marginal (11.2 to 13.3 *μ*mol/L). Only on sampling day 7, the difference between the proved lactations was significant (*P* < .05). 

The mean zinc concentration was significantly higher for cows staying in the second lactation (14.2 *μ*mol/L) in comparison to the first lactating cows (12.8 *μ*mol/L). During the whole experimental period only in Lactation 1 was the serum zinc concentration nearly constant (12.2–13.4 *μ*mol/L), whereas the serum zinc concentration in the second lactation was significantly higher at the start of the experimental feeding (16.5 to 12.4 *μ*mol/L), seven days after the beginning (15.7 to 13.0 *μ*mol/L) and at day 112 (14.7 to 13.0 *μ*mol/L, Figure 2(b)).

**Figure 3:**
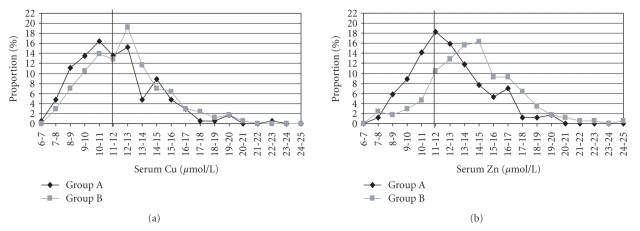
Distribution of all analysed serum copper (a) and zinc concentrations (b) in the different feeding groups (A and B).

The distribution of all analysed copper and zinc concentrations in the serum is demonstrated for the different feeding groups (A and B) in Figures 3(a) and 3(b). There is a significant difference in the distribution of all analysed serum copper concentrations (Figure 3(a)). The highest percentages (16% in Group A and 19% in Group B) were analysed between 10 and 11 *μ*mol/L (Group A), 12 and 13 *μ*mol/L (Group B). The total number of serum copper concentrations below the reference value (12 *μ*mol/L [19]) is significantly lower for Group B (47%) with a higher copper supplementation than for the cows in Group A (61%). 

This effect is much more pronounced for zinc (Figure 3(b)). The highest percentage (16%) of all analysed serum zinc concentrations is registered with 14-15 *μ*mol/L in Group B, whereas the highest percentage (18%) in Group A is analysed only between 11 and 12 *μ*mol/L; the distribution curve of the zinc concentrations in Group B moved with its maximum to the right side of the figure in comparison to the distribution curve of zinc concentrations in Group A. Consequently, the total number of serum zinc concentrations below the reference range (<12 *μ*mol/L [19]) is significantly lower in Group B (22%), whereas in Group A nearly half of all analysed zinc concentrations (48%) is below 12 *μ*mol/L.

**Figure 4:**
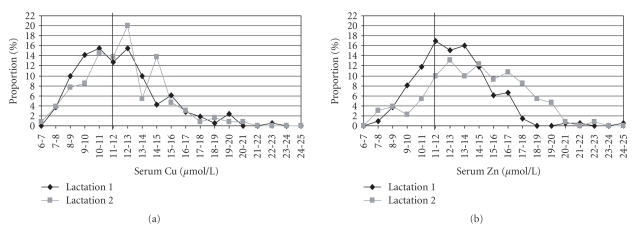
Distribution of all analysed serum copper (a) and zinc concentrations (b) in the two lactations (1 and 2).

The influence of the different lactations is demonstrated in Figures 4(a) and 4(b). The highest percentage of analysed copper concentrations (16%) is in a range between 10-11 and 12-13 *μ*mol/L in the first lactation, whereas the highest percentage of analysed copper concentrations (20%) in the second lactation was registered only in a tendentially higher range (12-13 *μ*mol/L). Between the two lactations, the number of serum copper concentrations below the minimum of the reference value (12 *μ*mol/L [19]) is not significantly different (56% in the first lactation and 50% in the second lactation, Figure 4(a)). However, the number of serum zinc concentrations lower than the minimum of the reference range (12 *μ*mol/L [19]) in total was lower than for copper in both lactations (42% in the first and 24% in the second lactation) and the percentage of low serum zinc concentrations is significantly reduced for cows staying in their second lactation. Consequently, the maxima of the curves of serum zinc concentrations (Figure 4(b)) moved to higher concentrations; the highest percentage of analysed zinc concentrations (17%) is in a range between 11-12 *μ*mol/L in the first lactation, whereas the highest percentage of analysed zinc concentrations in the second lactation (13%) was only in a slightly higher range (12-13 *μ*mol/L). 

### 3.3. Reference Values for Copper and Zinc

The calculated reference values for copper are not significantly influenced whether by the feeding group nor by the lactation number (Table 4). The calculated reference ranges for copper did also not significantly differ between the two different methods of calculation (parametric or nonparametric); the mean reference range for copper calculated with the parametric method is 7–17 *μ*mol/L, whereas the reference range for copper calculated with the nonparametric method is only tendentially higher (8–19 *μ*mol/L).

The calculated reference ranges for zinc are also not significantly different between the two calculation methods (parametric or nonparametric calculation). The mean reference range for zinc is 8–19 *μ*mol/L for both calculation methods. However, the reference ranges for zinc are tendentially higher for Group B and Lactation 2 in comparison to Group A and Lactation 1 (Table 5). 

## 4. Discussion

The different copper supply did not significantly influence the serum copper concentration. The serum copper concentration of the cows in Group B fed the double amount of copper (22.6 mg/kg DM) as that of the cows in Group A (11.1 mg/kg DM) was not significantly higher (12.3 *μ*mol/L to 11.7 *μ*mol/L) during the whole experimental feeding (252 days). These observations are in good agreement with other results [22–25]. However, the analysed serum copper concentrations in the study by Engle et al. [24] are higher (16.8 and 16.5 *μ*mol/L). All of these studies verified the thesis that no significant correlation exists between copper intake and copper concentration in serum when the cows were fed as recommended. Copper concentrations in the serum of cows are not indicative of levels provided in the diet and suggest that the cows are neither deficient nor received copper in amounts to be considered excessive [24]. In other studies, a higher copper supply resulted in higher copper concentrations in serum [26–28], but all the investigated copper contents (40, 50, and 60 mg/kg DM) were higher than the recommendations (10 mg/kg DM [12]) and are above the European upper level for copper (35 mg/kg DM [29]). A reason for the not significantly different serum copper concentrations could be the copper metabolism in combination with the recommended feeding in the present investigation. Feeding copper as recommended resulted in liver copper contents, which were higher than the reference ranges [30] with the consequence that there exists no copper deficiency. A copper deficiency (undersupply with copper) would react in a higher copper metabolising from the liver into blood. Extremely high copper supplementations [26–28] could result in a repletion of the liver, the overdosed copper is then absorbed and resulted in an increasing of copper in blood serum. 

A trend to an influence of copper supply on the distribution of serum copper concentrations was observed, because the percentage of serum copper concentrations lower than the reference values (<12 *μ*mol/L [19]) was tendentially reduced (47%) for Group B (receiving higher amounts of copper), whereas in Group A, 61% of all analysed serum copper concentrations were below 12 *μ*mol/L (Figure 3(a)). These results are comparable with the investigations of Laven and Livesey [27], who found a higher percentage of serum copper concentrations below 9 *μ*mol/L (22%) after a supplementation of 300 mg Cu/d. A dramatically higher copper supply than recommended (here 1250 mg Cu/d, which results in a copper content of approximately 60 mg/kg DM in the ration) resulted in only 3% of serum copper concentrations lower than 9 *μ*mol/L. The influence of the lactation number is not significant; the percentage of serum copper concentrations below 12 *μ*mol/L is also high in both lactations (56% of all copper concentrations in the first lactation and 50% in the second lactation are lower than 12 *μ*mol/L). Du et al. [23] and Laven and Livesey [27] found no significant differences in serum copper concentration between heifers and lactating cows.

In contrast to copper, the higher zinc supplementation (113.0 mg Zn/kg DM in Group B) resulted in significantly higher serum zinc concentrations on various sampling times (Days 28, 56, and 112). Additionally, the average zinc concentration during the whole experimental feeding is significantly higher for the animals in Group B (14.1 *μ*mol/L) in comparison to 12.5 *μ*mol/L for the cows in Group A. In other experimental studies with calves Kincaid et al. [31] have verified only tendentially higher serum zinc concentrations (19.6 *μ*mol/L) after a zinc supplementation of 300 mg/kg DM compared with 16.1 *μ*mol/L for unsupplemented calves. But this extremely high zinc content of 300 mg/kg DM is higher than the allowed upper level for zinc in the EU (150 mg/kg DM [29]). In another study, the serum zinc concentration increased significantly from 17.3 to 28.2 *μ*mol/L only after feeding extremely high zinc amounts (500 mg/kg DM [32]), whereas a lower zinc supplementation of 20 mg/kg DM to a basal diet containing 28 mg Zn/kg did not influence the plasma zinc concentration (16.8 *μ*mol/L and 16.2 *μ*mol/L). Nunnery et al. [33] found no significant differences in serum zinc concentrations (21.6 and 22.0 *μ*mol/L) after feeding 82 mg Zn/kg DM over 168 days in comparison to a control group (51 mg Zn/kg DM). All these experimental studies confirmed the thesis that the analysing of serum or plasma zinc concentration could only be a helpful parameter in diagnosing a sufficient zinc supply. But the results in total are very inhomogeneous, because the ingested zinc is distributed in various viscera and is not present in the blood [34].

The different zinc supply has a significant influence on the distribution of serum zinc concentrations. The percentage of serum zinc concentrations lower than the reference values (<12 *μ*mol/L [19]) was significantly lower (22%) for Group B, whereas in Group A, 48% of all analysed serum zinc concentrations were below 12 *μ*mol/L (Figure 3(b)). Also the lactation number had a significant influence on the distribution of analysed serum zinc concentrations (42% of all serum zinc concentrations in the first lactation, but only 24% in the second lactation are lower than 12 *μ*mol/L). The number of studies analysing the distribution of serum zinc concentrations after different zinc supplementation is very small in the literature. Meglia et al. [35] have detected that 63% of all analysed serum zinc concentrations were lower than 12 *μ*mol/L. However, in this study no data about the zinc content in the fed ration were given, and only the influence of calving stress on different mineral concentrations in blood serum was proven. 

The calculated reference ranges for copper (8–19 *μ*mol/L) as well as for zinc (8–19 *μ*mol/L) concentration in the serum of dairy cows in the present investigation are lower than the most reference values given in the literature. 

However, the reference ranges used in practice are often broad and their diagnostic performance is poor, probably as a result of methodological differences, because the analytical methods are usually not specified [36] and the suppositions for calculating reference ranges are unknown or not described exactly. But it is essential to have a standardised population of animals for the calculation of reference values (e.g., breed, sex, age, husbandry and feeding), if possible with consistent health and nutrition status [37]. The IFCC [7, 8, 38, 39] has recommended that reference ranges should be derived from a random sample of a clearly defined reference population of healthy individuals of the same species, living under comparable conditions and determined by the same, specified methods; any experimental protocol should minimise the effects of environmental influences and sampling techniques, and factors such as biological variation that cannot be influenced should be taken into consideration and evaluated. All these conditions are comprehensible in the present investigation, especially an exact description of the animals and feeding used. The recommendations of copper and zinc supply of the GfE [12] or NRC [40] are regularly adapted to the changing performance of dairy cows in the last ten years, but most reference ranges for trace element concentrations in serum were never changed or adapted. Are there other legitimacies for reference values than for the recommendations or must the reference ranges be evaluated more critically and eventually adapted to the current recommendations? A serum copper and zinc concentration of 8–19 *μ*mol/L could reflect a sufficient supply of dairy cows with these trace elements. For diagnosing a trace element deficiency, which is very important in veterinary practice, it is absolutely necessary to know “normal serum trace element concentrations”, which are derived under actual feeding conditions.

## 5. Conclusions

Copper and zinc analyses in blood serum are not qualified indicators to characterize the level of copper or zinc for dairy cows, when the cows were fed as recommended. Only an extremely overdosing of copper and zinc above the European upper levels resulted in a significant increase of serum concentrations. Other sources such as feedstuffs, liver, and cow hair must also be investigated for an exact diagnosis of trace element supply. A sufficient supply with copper and zinc only resulted in serum concentrations below the reference ranges, but without any significance of a clinical deficiency for the animals. The recommended reference values in blood serum, which reflect at least a sufficient supply with copper and zinc, followed by the present investigations (under standardised feeding conditions) are 8–19 *μ*mol/L for both trace elements. The applied reference ranges in practice must certainly be analysed in a more critical way and consequently adapted to the current feeding recommendations.

## Figures and Tables

**Table 1:** Different reference values for copper and zinc in blood serum in the literature.

Cu (*μ*mol/L)	Zn (*μ*mol/L)	Reference
16–32	—	[13]
12–20	>7.7	[14]
>12.6	>12.6	[15]
5.2–5.5	—	[16]
16–32	10.7–19.9	[17]
12.5–32.8	12–46	[18]
12–24	12–24	[19]
8–39	10–20	[20]
14–19	14.5–20.0	[21]

**Table 2:** Mean copper and zinc contents of the different feed components (mg/kg DM).

	Cu	Zn
Grass silage (*n* = 10)	8.2 ± 1.4	34.0 ± 5.9
Maize silage (*n* = 10)	4.8 ± 0.5	23.7 ± 2.6
Concentrate A (*n* = 9)	18.2 ± 0.5	105.5 ± 2.9
Concentrate B (*n* = 9)	47.3 ± 1.4	243.3 ± 7.1
Total ration A (*n* = 9)	11.1 ± 1.6	61.9 ± 17.0
Total ration B (*n* = 9)	22.6 ± 4.9	113.0 ± 30.0

**Table 3:** Mean dry matter (DM)-intake (kg/d) as well as copper and zinc intake (mg/d).

	DM-intake	Copper-intake	Zinc-intake
Group A (*n* = 30)	17.3 ± 3.3	193^a^ ± 39	1041^a^ ± 215
Group B (*n* = 30)	17.4 ± 3.2	395^b^ ± 78	1993^b^ ± 397
Lactation 1 (*n* = 38)	16.4^a^ ± 2.9	277 ± 111	1428 ± 540
Lactation 2 (*n* = 22)	19.1^b^ ± 3.1	322 ± 128	1662 ± 622

a < b; *P* < .05.

**Table 4:** Calculated reference ranges for copper (*μ*mol/L) in dependence on the different feeding groups (A and B) and lactations (1 and 2).

	Parametric calculation	Non parametric calculation
Group A (*n* = 170)	6–17	8–18
Group B (*n* = 172)	7–18	8–19
Lactation 1 (*n* = 212)	6–17	8–19
Lactation 2 (*n* = 130)	7–17	8–18

**Table 5:** Calculated reference ranges for zinc (*μ*mol/L) in dependence on the different feeding groups (A and B) and lactations (1 and 2).

	Parametric calculation	Non parametric calculation
Group A (*n* = 170)	7–18	9–18
Group B (*n* = 172)	8–20	8–20
Lactation 1 (*n* = 212)	8–18	9–17
Lactation 2 (*n* = 130)	8–21	8–20
